# Case of Placenta Previa

**Published:** 1846-12

**Authors:** W. Butterfield

**Affiliations:** Ottawa, Ill.


### ARTICLE III.

*Case of Placenta Previa.* By W. Butterfield, M. D., Ottawa, Ill.

The following case will perhaps be considered somewhat illustrative of the "vis medicatrix naturæ," upon a point of great importance in obstetric practice, which has lately occasioned a spirited controversy between high authorities. Taken as an isolated case, it would seem to sanction the practice of removing the placenta, where flooding becomes dangerous, before the delivery of the child is accomplished either naturally or by turning, as the most effectual means of arresting hemorrhage in placenta previa. On the 20th of May, last, early in the morning, I was requested to visit Mrs. I., in labor with her second child. I was informed that she considered herself about seven months advanced in pregnancy. It appeared that upon rising from her bed a short time previously, she was seized with severe pain which was followed by the rupture of membranes with escape of the waters. The pains occurred at short intervals accompanied by hemorrhage. On examination, I found the os uteri dilated to about the size of a quarter of a dollar, but exceedingly rigid and resisting: nevertheless, I satisfied myself that it was a case of placental presentation. During each pain the funis in which no pulsation was perceptible protuded into the vagina. After waiting a considerable time, as the pains did not increase either in strength or frequency, and the hemorrhage being but trifling, I determined to leave, enjoining my patient to observe strict rest and the horizontal posture during my absence. The room was ordered to be kept cool, and acidulated beverage administered. The T. opii. camph. was also given more for the purpose of calming and soothing the patient's mind than on account of its medicinal properties. On returning after a lapse of three hours, the patient was found in the same condition and therefore interference was neither necessary, nor practicable. After repeating my injunctions as to rest and the posture, and requesting I might be informed the instant either flooding or pain returned, I again left. At the expiration of four hours, not having received a message in the interim, I paid another visit. The os uteri was now more dilated and less rigid, with a considerable segment of the placenta projecting into the vagina. As however the uterine action was still feeble, the hemorrhage but trifling and the patient's spirits and strength being good, with an unblanched countenance, I determined upon a further delay. I therefore took my departure with a distinct understanding that I should be apprised as soon as any emergency arose. About half past nine o'clock P. M., I received an urgent summons to attend. On my arrival, the placenta had just been expelled, and both arms and one leg of the fœtus were protuding through the os externum. In this state of things, the obvious course was to bring down the inferior extremities, an object I was preparing to accomplish, when a violent pain came on, which suddenly effected the delivery without any alteration having occurred in the presenting position of the child. The hemorrhage entirely ceased on the expulsion of the placenta, and the patient experienced an uninterrupted and a rapid recovery. It is almost needless to add, that the child, apparently of about seven month's growth, was dead.

